# Edge-Driven Disability Detection and Outcome Measurement in IoMT Healthcare for Assistive Technology

**DOI:** 10.3390/bioengineering12101013

**Published:** 2025-09-23

**Authors:** Malak Alamri, Khalid Haseeb, Mamoona Humayun, Menwa Alshammeri, Ghadah Naif Alwakid, Naeem Ramzan

**Affiliations:** 1 Department of Computer Science, College of Computer and Information Sciences, Jouf University, Sakaka 72311, Saudi Arabiagnalwakid@ju.edu.sa (G.N.A.); 2 Research and Innovation Groups, King Salman Center for Disability Research, Riyadh 11614, Saudi Arabia; 3 Department of Computer Science, Islamia College Peshawar, Peshawar 25120, Pakistan; khalid.haseeb@icp.edu.pk; 4 School of Computing, Engineering and the Built Environment, University of Roehampton, London SW15 5PU, UK; mamoona.humayun@roehampton.ac.uk; 5 School of Computing, Engineering and Physical Sciences, University of West of Scotland Paisley, Paisley PA1 2BE, UK; naeem.ramzan@uws.ac.uk

**Keywords:** disability detection, edge computing, healthcare application, internet of things, assistive technology

## Abstract

The integration of edge computing (EC) and Internet of Medical Things (IoMT) technologies facilitates the development of adaptive healthcare systems that significantly improve the accessibility and monitoring of individuals with disabilities. By enabling real-time disease identification and reducing response times, this architecture supports personalized healthcare solutions for those with chronic conditions or mobility impairments. The inclusion of untrusted devices leads to communication delays and enhances the security risks for medical applications. Therefore, this research presents a Trust-Driven Disability-Detection Model Using Secured Random Forest Classification (TTDD-SRF) to address the issues while monitoring real-time health records. It also increases the detection of abnormal movement patterns to highlight the indication of disability using edge-driven communication. The TTDD-SRF model improves the classification accuracy of abnormal motion detection while ensuring data reliability through trust scores computed at the edge level. Such a paradigm decreases the ratio of false positives and enhances decision-making accuracy in coping with health-related applications, mainly the detection of patients’ disabilities. The experimental analysis of the proposed TTDD-SRF model indicates improved performance in terms of network throughput by 48%, system resilience by 42%, device integrity by 49%, and energy consumption by 45% while highlighting the potential of medical systems using edge technologies, advancing assistive technology for healthcare accessibility.

## 1. Introduction

The rapid growth and advancement of healthcare systems utilizing the IoMT enable continuous and remote monitoring of individuals, particularly those with disabilities [1,2]. These systems integrate cutting-edge technologies and wearable devices that capture vital physiological and motion data, facilitating the early detection of health anomalies and supporting decision-making in assistive healthcare paradigms. IoMT devices generate vast amounts of physiological and motion-related data from patients, including individuals with mobility impairments, creating big data that must be processed in real time for effective healthcare applications [3,4]. In critical systems, efficient analysis of these data is essential for ensuring accessibility, maintaining system reliability, and reducing response time, particularly in detecting disabilities and abnormal movement patterns [5,6]. The increasing demand for real-time data processing in healthcare systems, particularly for individuals with disabilities, has highlighted the limitations of traditional cloud computing approaches. These methods struggle with issues such as data latency and the efficient utilization of bandwidth and energy, especially when dealing with battery-powered wearable sensors [7,8]. To overcome these challenges, the integration of edge computing in healthcare environments offers a promising solution. Edge computing enables localized data preprocessing near the source devices, significantly reducing response time and enhancing system availability during critical decision-making processes [9,10]. With the combination of intelligent devices, most smart systems can identify diseases more quickly and reliably for constrained applications. However, many existing schemes overlook trustworthiness while transmitting health data in an unpredictable healthcare environment, which increases the additional overhead on the performance of the developed system [11,12,13].

On the other hand, if sensitive health data are captured and compromised, it leads to violations of confidentiality and integrity. It decreases the accuracy of disease detection based on actual and received data at network edges [14,15]. Security in healthcare applications plays a critical role in protecting sensitive patient data and real-time collected physiological signals using wearable smart devices [16,17]. If security is compromised, malicious access may lead to increased misdiagnosis, thereby affecting the effectiveness of the medical system through incorrect treatment decisions [18,19]. In addition to these considerations, the electromagnetic safety of IoMT devices is an increasingly important aspect that must be addressed. As wearable devices emit electromagnetic radiation, it is crucial to monitor the Specific Absorption Rate (SAR) and temperature variations to ensure that the levels remain within safe limits when interacting with human tissues. Recent studies have highlighted the need for careful management of electromagnetic exposure to prevent potential harm to users. Thus, in addition to optimizing performance, IoMT monitoring systems must also consider electromagnetic safety to ensure patient well-being during real-time health monitoring. This broadens the scope of disability detection to include critical safety aspects, ensuring that the devices not only perform effectively but also adhere to safety standards. The proposed model aims to design and develop an intelligent monitoring system for disability detection that combines edge computing. The IoMT wearable sensors collect and transmit medical signals from the patient’s body to identify abnormal motion patterns. It explores multi-feature analysis with the support of the Random Forest classifier [20] and incorporates trust evaluation methods to enhance the authenticity of the medical system. Our proposed model also reduces false positives, thereby enhancing clinical decision-making using edge-driven computing.

### Contributions

Our proposed work focuses on developing a lightweight edge-based healthcare solution to provide a real-time disability-detection model while attaining high precision and security. It leads to the contribution of adaptive healthcare systems for reliably analyzing health conditions. In addition, by incorporating these advancements, the TRDD-SRF model can be adapted for real-time monitoring in assistive telemedicine systems, where continuous data acquisition from wearable sensors would allow for timely feedback and decision-making in a home-based healthcare environment. Such potential extends our model to home monitoring applications and highlights its broader relevance for telemedicine and personalized healthcare. The major contributions of our research work are highlighted as follows.

- It integrates wearable IoMT sensors with edge computing to develop a timely algorithm for disability detection. By combining motion data and sEMG signals, the Random Forest classifier extracts key features to distinguish between normal and abnormal movement patterns, enhancing the real-time diagnostic capabilities of assistive healthcare systems.
- Trust integration is employed to ensure the reliability of data from IoMT devices, thereby improving the accuracy of disability detection and disease classification. This approach reduces false positives and enhances decision-making strategies by filtering out unreliable data, ensuring that healthcare systems can provide trustworthy assessments for individuals with disabilities.
- The use of edge computing for preprocessing data minimizes computational overhead on IoMT devices, enabling an energy-efficient and low-latency communication system. This optimization is crucial for real-time healthcare applications, particularly in systems designed to monitor and assist individuals with disabilities.

The following sections comprise this research work: Section 2 presents the related discussion. Section 3 discusses the proposed model. Section 4 describes the experimental results with state-of-the-art schemes. Finally, Section 5 concludes the paper.

## 2. Related Work

The growing integration of tiny medical devices and lightweight edge computing has provided an extension of research growth for healthcare monitoring systems. They generate a massive amount of medical data and process it with advanced technologies for the betterment of human lives [21,22]. Before edge computing, constrained applications were significantly impacted by the excessive use of network resources in decision-oriented medical services. Additionally, most existing schemes have faced numerous challenges in identifying diseases promptly and reducing system availability during critical times [23,24]. While centralized processing used cloud systems, traditional applications required high computational power and were limited by data latency, making them unsuitable for time-constrained healthcare applications that need to identify diseases promptly [25,26].

Recent advancements in structural fault detection for biomedical devices have explored the use of non-invasive techniques to detect defects such as delamination in biomedical composites. One promising approach involves the integration of soft computing models with classifiers to estimate and classify defects commonly used in biomedical devices [27,28]. These techniques leverage edge-based anomaly detection, enabling real-time, efficient detection and classification of structural defects.

By combining soft computing approaches with classifiers, researchers have improved the accuracy and reliability of non-invasive defect detection methods, ensuring enhanced safety in biomedical applications [29,30]. Moreover, recent studies have addressed the development of home care devices and patient monitoring systems, which integrate wearable sensors for continuous health tracking in non-clinical environments [31,32]. One notable advancement is the development of sensorized non-invasive ventilators for telemedicine applications, enabling remote monitoring and healthcare delivery. These systems, which rely on real-time data transmission, share similarities with our proposed approach of using IoMT sensors for disability detection. In [33], authors proposed a hybrid and robust priority-based Duty-Cycled with Ant Colony Optimization Routing (DC-ACOP) mechanism. It effectively utilizes the resources of IoT systems, illustrating improved energy efficiency through the timely delivery of packets in Smart Healthcare Systems (SHS). The proposed solution ensures the transmission of critical health data with minimal delay, leveraging a combination of priority-based duty cycling and an enhanced Ant Colony Optimization (ACO) routing strategy. The performance results are obtained through simulations that enhance network lifetime, energy efficiency, and packet delivery ratio, particularly for critical health data. A combined approach with a feature-driven FCNN classification model is proposed by [34] that improves the accuracy and efficiency of multimodal healthcare analysis. The proposed system achieves robust classification of neurological or motor disorders using preprocessing and extracting features from both voice signals and handwriting spiral images. The experimental results demonstrate its optimized routing efficiency, latency, and identification methods for real-time monitoring applications in the medical sector.

In [35], using enhanced mayfly clustering-based Q learner routing (EMCQLR) and exponential key-based elliptical curve cryptography (EKECC) techniques, the authors proposed a secure and energy-efficient data transmission framework for IoT-based HC (IoT-HC) systems. Initially, double hash biometric-based authentication (DHABA) is utilized to authenticate IoT users and prevent the network from having vulnerable access. Using the enhanced mayfly optimization algorithm (EMOA), the cluster head is selected, and various clusters are formed for IoT sensors. To attain reliable routing, duplication is first checked, and then the path-weighted Q reinforcement learning (PWQRL) model is explored. Authors [36] introduced the Healthcare System using Private Blockchain-based Cloud IoT (HSPBCI) services. It protects against data compromise and maintains authorized access for IoT data against threats. The primary objective of the proposed framework is to secure block-based transactions and minimize encryption and decryption times/decryption time with authorized access to health records. Only devices with the appropriate data keys are permitted to access the patient’s data, ensuring support for data privacy. Moreover, a cloud blockchain network is used to filter out suspicious users and service providers, ensuring a reliable healthcare system.

In [37], authors present a hybrid framework for upper limb rehabilitation, combining finite element modeling (FEM), AI-based trend classification, and a custom-designed electronic system for real-time signal acquisition and wireless data transmission. A COMSOL 6.2 Multiphysics model simulates the interaction between a robotic glove and a deformable latex sphere, using a Mooney–Rivlin hyperelastic formulation for large nonlinear deformations. High-fidelity simulation data are used to train an AI algorithm that classifies rehabilitation progress. The integrated electronic system supports real-time feedback and cloud-based monitoring, demonstrating robust performance in rehabilitation settings. Authors in [38] introduced a Cross-Layer and Cryptography-based Secure Routing (CLCSR) protocol to enhance security and data privacy levels in healthcare applications using a two-phase approach. To provide security against multi-layer attacks, the first phase focuses on cross-layer secure clustering with the optimal selection of cluster heads using a probabilistic model. On the other hand, the second phase guarantees the device’s privacy and authorized data access using lightweight Elliptic Curve Cryptography (ECC). It enhances the threat detection methods and provides secure healthcare transmissions in the presence of malicious activities. Table 1 summarizes key existing approaches in healthcare monitoring and their limitations, along with the main contributions of our proposed TRDD-SRF model in enhancing real-time disability detection and system efficiency.

### Problem Formulation

As discussed, despite significant advances in health monitoring, many existing approaches utilizing AI-driven techniques have focused solely on single-modal data. Moreover, most related solutions have overlooked privacy concerns and lack trusted methods for processing medical data, which limits the accuracy of real-time monitoring systems and reduces confidence in early-stage disability detection. Additionally, many proposed solutions fail to effectively manage resources in healthcare processing and decision-making, diminishing their impact on critical applications. To address these challenges, we propose an optimized and reliable healthcare multi-phase framework that enhances the accuracy of disability detection. This framework integrates trust-aware wireless computing collaboration, offering a timely decision-making system and improving security against potential threats. Furthermore, we integrate soft computing approaches with our research on disability detection using IoMT devices. In a similar way to edge-based anomaly detection used for real-time defect detection in biomedical devices, we apply these techniques to monitor movement patterns in patients. By utilizing Random Forest and edge computing, our work ensures both the accuracy of disability detection and the safety of patients, providing real-time feedback in healthcare applications.

## 3. Materials and Methods

This study proposes a trust-aware health monitoring framework that integrates IoMT and lightweight edge computing for real-time disability detection. Multiple criteria-based features are extracted using health sensors to identify the human body’s motion patterns. A Random Forest Classifier is explored to identify the patterns between normal and abnormal classes. Additionally, a trust-driven evaluation is applied to untrusted devices to ensure reliable decision-making based on collected medical data. The proposed model leverages the processing capabilities of edge computing for data aggregation and processing, while also ensuring efficient management of system resources in critical medical applications. The proposed TRDD-SRF model is composed of three main phases. The first phase addresses sensor-based data acquisition and lightweight, edge-driven preprocessing. Wearable health devices are used to observe and collect raw motion and physiological data from patients’ bodies. Continuous observation of a patient’s body enables the timely and real-time analysis of body movement, allowing for the identification of patterns of disability. Health sensors *S* collect samples of motion data at time interval *t* using Equation (1).

(1) $$S\left(t\right)=\left[\begin{array}{c} a_{x}\left(t\right)\\ a_{y}\left(t\right)\\ a_{z}\left(t\right)\\ \omega_{x}\left(t\right)\\ \omega_{y}\left(t\right)\\ \omega_{z}\left(t\right)\\ \theta (t) \end{array}\right]$$

where *a_x_*, *a_y_*, *a_z_* denotes linear acceleration, *ω_x_*, *ω_y_*, *ω_z_* are angular velocity, and θ(t) is postural angle. Later, a noise filtering technique is applied to the collected health data to produce error-free and normalized data for feature extraction, trust evaluation, and disability detection. Each collected sensor data *S* at time interval *t* is incrementally averaged using a recursive moving average filter over a window of size *w*, as given in Equation (2).

(2) $$\bar{S}\left(t\right)=\bar{S}\left(t-1\right)+\frac{1}{w}\left[S(t)-S(t-w)\right]$$

After the preprocessing stage, various features are extracted to identify movement patterns as normal or abnormal, as outlined in Equations (3)–(5).

Equation (3) computes the acceleration magnitude from its components along the *x*, *y*, and *z* axes. It is mainly utilized to detect patient movement patterns.

(3) $$a\left(t\right)=\sqrt{\vec{a}\left(t\right)\cdot \vec{a}\left(t\right)}$$

The angular velocity denotes the change in rotational movement of the patient’s body at time *t* around the *x*, *y*, and *z* axes, and can be computed in Equation (4).

(4) $$\omega \left(t\right)=\sqrt{{\left[\begin{array}{ccc} \omega_{x}\left(t\right) & \omega_{y}\left(t\right) & \omega_{z}\left(t\right) \end{array}\right]}^{T}\cdot \left[\begin{array}{c} \omega_{x}\left(t\right)\\ \omega_{y}\left(t\right)\\ \omega_{z}\left(t\right) \end{array}\right]}$$

Equation (5) identifies the movement analysis in posture detection and computes the patient’s body orientation angle from accelerometer data.

(5) $$\theta \left(t\right)=\arctan\left(\frac{\sqrt{a_{x}^{2}\left(t\right)+a_{z}^{2}\left(t\right)}}{a_{y}\left(t\right)}\right)$$

To incorporate the sEMG signal, the Root Mean Square (RMS) of the sEMG signal is computed over a time window *t* as shown in Equation (6).

(6) $$EMG\left(t\right)=\sqrt{\frac{1}{N}\sum_{i=1}^{N}{\left|EMG(t_{i})\right|}^{2}}$$

where $EMG(t_{i})$ represents the sEMG signal at time $t_{i}$, and *N* is the number of samples within the window. Alternatively, the Mean Absolute Value (MAV) of the sEMG signal can be computed using Equation (7).

(7) $$EMG\left(t\right)=\frac{1}{N}\sum_{i=1}^{N}\left|EMG(t_{i})\right|$$

where $EMG(t_{i})$ is the sEMG signal at time $t_{i}$, and *N* is the number of samples in the window.

After extracting the selected motion features and sEMG signals from the health sensors, the proposed TRDD-SRF model computes the weighted score $W_{\mathrm{score}}$, based on the significance of each feature in detecting abnormal movement patterns, as defined in Equation (8). The features include motion data and muscle activity, with each feature weighted to reflect its contribution to the movement analysis.

(8) $$W_{\mathrm{score}}=w_{1}\cdot a\left(t\right)+w_{2}\cdot \omega \left(t\right)+w_{3}\cdot \theta \left(t\right)+w_{4}\cdot EMG\left(t\right)$$

where $w_{1}$, $w_{2}$, $w_{3}$, and $w_{4}$ denote the weights assigned to the extracted features, including motion sensor data (a(t), ω(t), θ(t)) and sEMG data (EMG(t)) in the movement analysis of the patient’s body.

The Random Forest Classifier is explored based on the computed weighted score and other sensor-enabled features to identify the movement class, distinguishing between normal and abnormal patterns. Equation (9) denotes the construction of the feature vector $X_{i}$ with the combination of a weighted score and a set of features.

(9) $$X_{i}=\left[W_{score},\;f^{\left(i\right)}\right]$$

where $f^{\left(i\right)}$ denotes the vector of additional features for the *i*-th sample.

Afterwards, a random forest classifier is applied to the computed feature vectors and trains the model to identify the normal and abnormal movement patterns of patients’ bodies. It is the combination of various decision trees $D_{T}$ that are applied on the feature vector $X_{i}$, and the final predicted label ${\hat{y}}^{\left(i\right)}$ is selected by computing the voting criteria vot, as defined in Equation (10).

(10) $${\hat{y}}^{\left(i\right)}=\mathrm{vot}\left\{h_{t}\left(X_{i}\right)\;|\;\begin{array}{c} t=1,\ldots ,T \end{array}\right\}$$

The proposed TRDD-SRF model utilizes edge computing to enable real-time data processing within healthcare systems, ensuring rapid response times essential for detecting abnormalities. By processing health data closer to the source devices, this approach reduces latency and enhances the decision-making process. This reduction is crucial for identifying abnormal movement patterns in patients. The latency for both edge and cloud-based data processing can be represented using Equation (11).

(11) $$\left[\begin{array}{c} {\mathrm{Latency}}_{\mathrm{edge}}\\ {\mathrm{Latency}}_{\mathrm{cloud}} \end{array}\right]=\left[\begin{array}{c} B_{\mathrm{edge}}\cdot HD+P_{\mathrm{edge}}\\ B_{\mathrm{cloud}}\cdot HD+P_{\mathrm{cloud}}+\delta \end{array}\right]$$

Here, $B_{\mathrm{edge}}$ and $B_{\mathrm{cloud}}$ represent the bandwidth of the edge and cloud networks, respectively. HD denotes the health data being transmitted, $P_{\mathrm{edge}}$ and $P_{\mathrm{cloud}}$ refer to the processing delays at the edge and cloud, respectively, and δ indicates the additional delay introduced during cloud processing.

These equations quantify the latency involved in edge and cloud computing systems. The edge model facilitates faster response times by reducing the transmission distance and computational delays, thereby improving the system’s ability to detect abnormalities and make timely decisions in healthcare applications. On the other hand, evaluating the trustworthiness of the proposed TRDD-SRF model guarantees that health data processing is more reliable and that only authentic devices are involved in the decision-making process. It depends on the computed trust score $T_{S}$, which uses accuracy $A_{i}$ and freshness $fr_{i}$ factors in a weighted manner, given in Equation (12). The higher the trust score, the more consistent the healthcare operation is in the unpredictable environment, and the more secure the communication for the crucial medical system.

(12) $$T_{i}=w_{1}\cdot A_{i}+w_{2}\cdot Fr_{i}$$

$A_{i}$ indicates the accurate level of health data in reflecting the actual movement of the patient *i*, defined in Equation (13) as the proportion of correct readings *n* to the total number of collected sensor readings $c_{ij}$.

(13) $$A_{i}=\frac{\sum_{j=1}^{n}c_{ij}}{\sum_{j=1}^{n}t_{ij}}$$

The timeliness of health data is crucial in the medical system, as outdated data may lead to inconsistent outcomes and incorrect identification of abnormal movements. Equation (14) defines the computation of $Fr_{i}$ using time variation Δt, and sensitivity coefficient α.

(14) $$T_{s}=1+\alpha \cdot \Delta t$$

**Figure 1:**
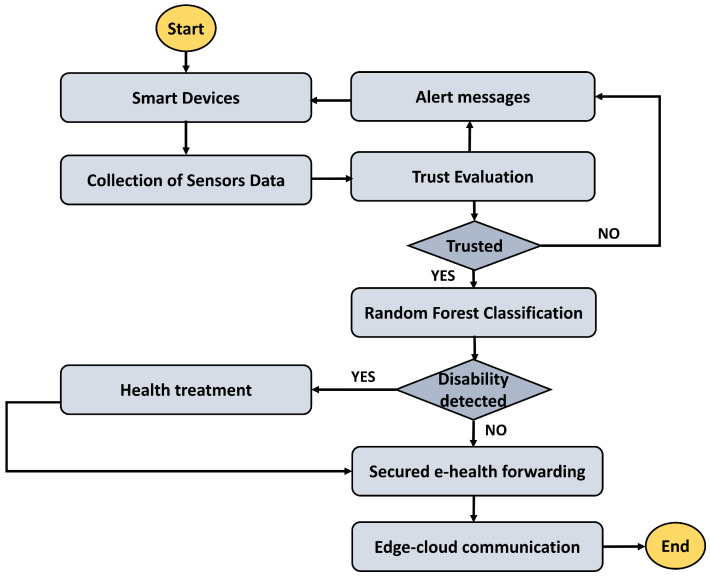
Working flow of the proposed TRDD-SRF model for Trust-driven AI disability detection.

The proposed TRDD-SRF model is designed in two main phases, as shown in Algorithm 1 for edge-level preprocessing and trust computing, and Algorithm 2 for random forest-driven disability classification. Firstly, the algorithm acquires continuous motion signals from wearable IoMT sensors and preprocesses them at the network edges using noise filtering techniques. This preprocessing ensures the effective utilization of healthcare resources with high energy efficiency and minimal latency, which is critical for real-time operations. Only the most relevant features among the collected ones are analyzed and forwarded for the decision-making process. This approach enhances the response time for medical treatment, allows for the timely identification of movement patterns, and improves the reliability of device interactions by incorporating trust-based computations. Secondly, a multimodal feature extraction process is applied to the preprocessed signals, where weighted scores are computed using features such as acceleration, angular velocity, and posture data. To ensure the reliability of health data transmission and accuracy in detecting disabilities, the proposed model incorporates trustworthiness computing, which weighs the accuracy and data freshness factors. The resulting weighted feature vectors are then provided as input to the Random Forest classifier, which classifies the motion patterns as either normal or abnormal, identifying potential disabilities in the patient’s movement. This classification is a crucial component of the proposed model for real-time healthcare applications, ensuring timely and accurate detection of abnormal movement patterns. Figure 1 depicts the working procedure of the proposed TRDD-SRF model using trust-driven AI disability detection for healthcare applications. The health devices continue to capture the patients’ conditions and interact with each other to analyze and transmit them to the edge nodes. The trust is computed for the authentication of health metrics and IoMT devices, and according to the announcement messages, they are declared. If any malicious activity is detected, the device is recorded in the memory table, and its data are excluded from the health diagnostic process. Our proposed TRDD-SRF model utilizes the random forest classification algorithm to predict and identify early signs of disability based on collected health records, thereby transmitting the information to processing systems for timely treatment.

**Algorithm 1:** Edge-Based Data Preprocessing with Trust-Driven Evaluation

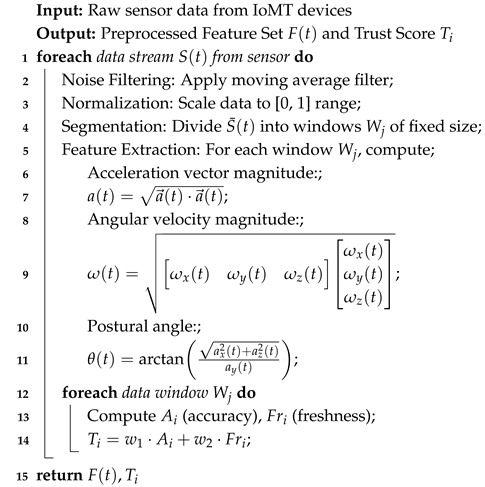



**Algorithm 2:** Random Forest-Based Multiple Features-Enabled Disability Detection

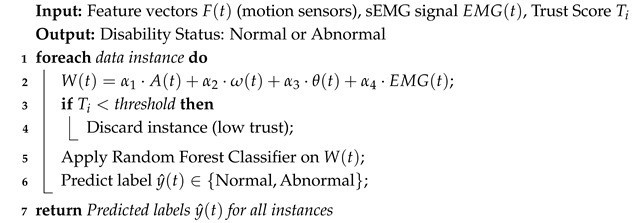



Our proposed TRDD-SRF model offers distinct key characteristics in the development of a smart healthcare system utilizing edge computing and intelligent learning methods. It includes data preprocessing, real-time classification for disability detection, and trust-driven communication. Table 2 presents the core features and characteristics of the proposed TRDD-SRF model, which ensures the trustworthiness management of health records and interaction between health devices.

Table 3 presents the security analysis for the proposed healthcare system, focusing on confidentiality and system resilience through the integration of edge-based preprocessing and trust-driven device evaluation. It offers a robust solution to mitigate security risks while enhancing the reliability and trustworthiness of disability detection in healthcare systems.

## 4. Simulation Environment

This section presents the simulation environment performance results for the proposed TRDD-SRF model and existing work, utilizing varying IoT devices and sampling rates in a smart healthcare environment. The simulated setting comprises 100–300 IoMT devices, including wearable sensors that monitor the patient’s body with varying data sizes based on their sampling rates. The deployed devices have limited constraints in terms of battery power, processing, and memory. The key metrics for the proposed TRDD-SRF model are examined, including network throughput, packet drop ratio, latency, and energy consumption. The UCI-HAR dataset [39] is used for performance evaluation. It contains sensor data collected from various participants who wore accelerometers and gyroscopes mounted on their waist. The data used in the simulation is based on the UCI-HAR, which was preprocessed and explored before being incorporated into the simulation model. The dataset includes time-series data with both time-domain and frequency-domain features, capturing various motion-related signals. The participants’ data were used for classification tasks to assess the proposed model’s accuracy and performance. The data are preprocessed at the network edges using trust-based weighted feature extractions to enhance the performance of the TRDD-SRF model. This preprocessing step ensures that only the most relevant and trustworthy features are used to optimize classification accuracy. The simulation was conducted on an Intel Core i7 processor with 32 GB of RAM, running Ubuntu Operating System. Performance metrics, including accuracy, precision, recall, and F1-score, are evaluated for assessing the proposed TRDD-SRF model. Table 4 shows the simulation parameters that are used in experiments.

### Results Discussion

**Figure 2:**
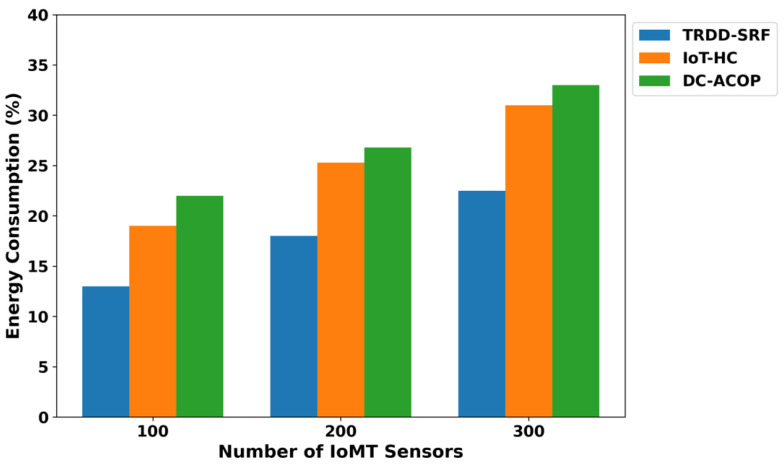
Energy consumption for varying IoMT sensors.

**Figure 3:**
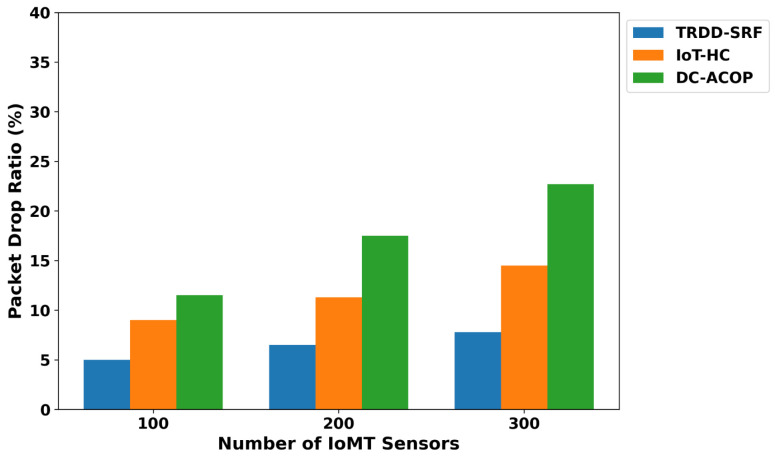
Packet drop ratio for varying IoMT sensors.

**Figure 4:**
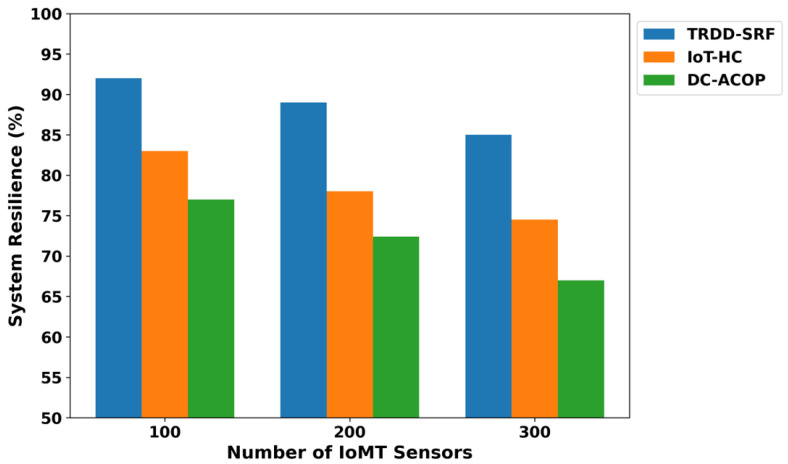
System resilience for varying IoMT sensors.

The TRDD-SRF model demonstrates a significant reduction in energy consumption, as shown in Figure 2. As compared to IoT-HC and DC-ACOP, the TRDD-SRF model improves the energy consumption by an average of 41.6% and 49%, respectively, across varying numbers of IoMT sensors. It is due to the inclusion of a trust-driven routing strategy with the support of edge computing, which prioritizes energy-efficient IoT routes and optimizes the resources of the healthcare environment. The TRDD-SRF model filters out congested links and avoids transmitting irrelevant healthcare records. In addition, the integration of edge computing further minimizes the load on the constrained devices and decreases the frequency of communication with the cloud platform. It not only provides a sustainable solution for the healthcare system but also efficiently utilizes the device consumption in a crucial environment. As depicted in Figure 3, the proposed TRDD-SRF model demonstrates significant performance in terms of packet drop ratio as compared to IoT-HC and DC-ACOP. The proposed model consistently improved the performance of the models over varying IoMT sensors by an average of 46% and 49%, respectively. It is due to the selection of more reliable forwarders while carrying health data towards the edges, and it increases the flow capacity of the IoT links. In addition, the trust is dynamically updated using multi-facet factors and enhances the leveraging of edge-based processing to minimize delays. Moreover, the reliable devices have been prioritized based on the Random Forest classifier, thereby enhancing the TRDD-SRF model’s ability to improve data delivery in a constrained environment. The improvement in terms of packet disturbance also demonstrates that the TRDD-SRF model is scalable, capable of efficiently processing critical healthcare data as the network size increases. The system resilience analysis, shown in Figure 4, demonstrates the TRDD-SRF model’s superior ability to maintain system stability during device failures or network disruptions. The proposed model consistently achieves resilience values that are 39% and 44% higher than those of existing approaches. This high resilience is attributed to edge-level processing, which enables critical healthcare decisions to be made locally, minimizing dependence on centralized cloud systems. Moreover, the trust-driven evaluation ensures that only reliable and secure devices contribute to the system, further strengthening the overall system stability and reliability.

**Figure 5:**
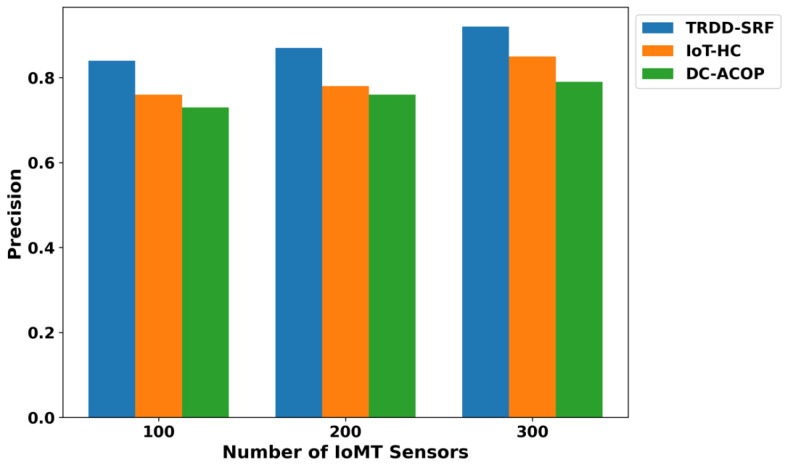
Evaluation of precision for TRDD-SRF, IoT-HC, and DC-ACOP.

**Figure 6:**
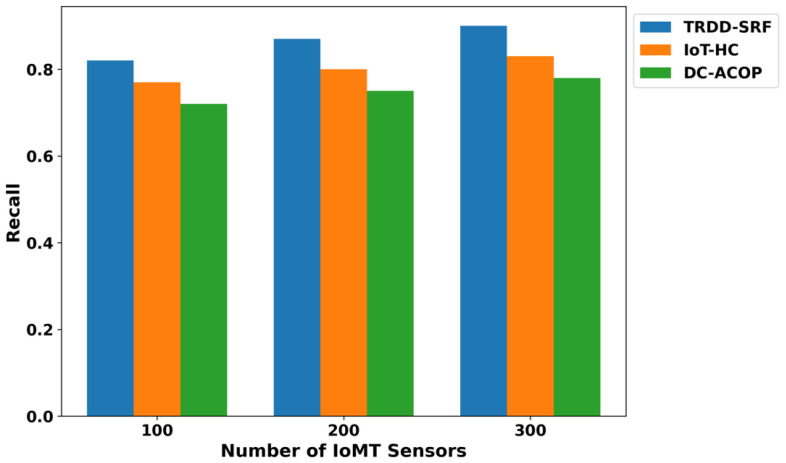
Evaluation of recall for TRDD-SRF, IoT-HC, and DC-ACOP.

**Figure 7:**
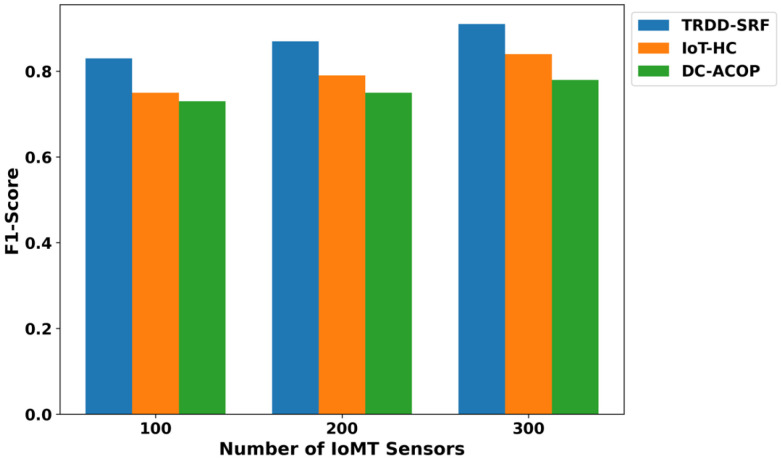
Evaluation of F1-score for TRDD-SRF, IoT-HC, and DC-ACOP.

**Figure 8:**
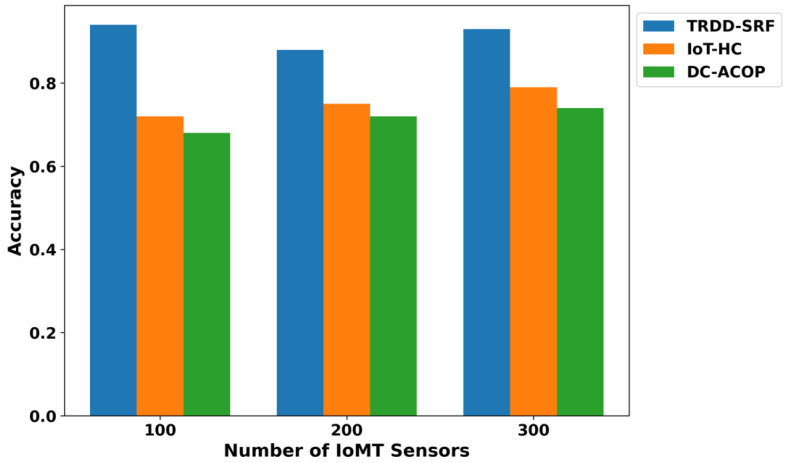
Evaluation of accuracy for TRDD-SRF, IoT-HC, and DC-ACOP.

The performance of the proposed TRDD-SRF model in terms of precision, recall, F1-score, and accuracy demonstrates significant improvements over existing approaches, as illustrated in Figure 5, Figure 6, Figure 7 and Figure 8. This improvement can be attributed to the trust-based feature extraction, which ensures that only reliable data are considered for decision-making. In terms of precision, the TRDD-SRF model shows a higher value, indicating its ability to correctly identify true positive instances without misclassifying normal movement as abnormal. The model also demonstrates a significant improvement in recall, ensuring that the majority of abnormal movements are detected, thereby minimizing false negatives. As for the F1-score, the TRDD-SRF model balances precision and recall effectively, with values consistently higher than those of IoT-HC and DC-ACOP. These metrics collectively indicate that the proposed model provides a more accurate, reliable, and effective solution for real-time disability detection in healthcare applications, especially when compared to existing approaches that rely on single-feature analysis or raw sensor data without considering trustworthiness.

**Figure 9:**
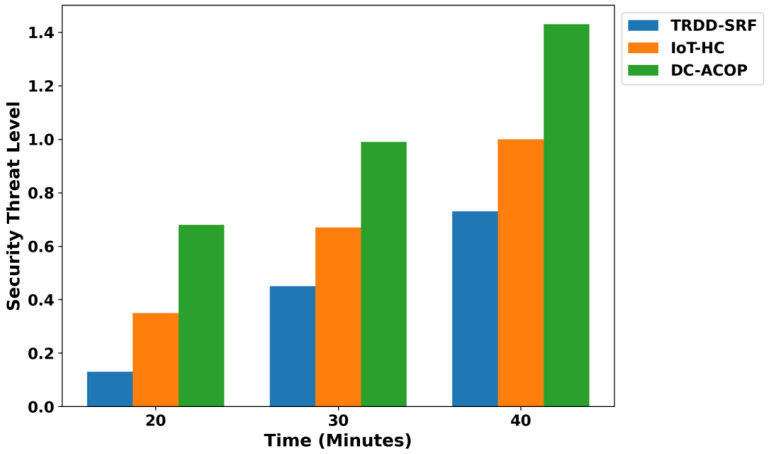
Trust score for varying health devices.

**Figure 10:**
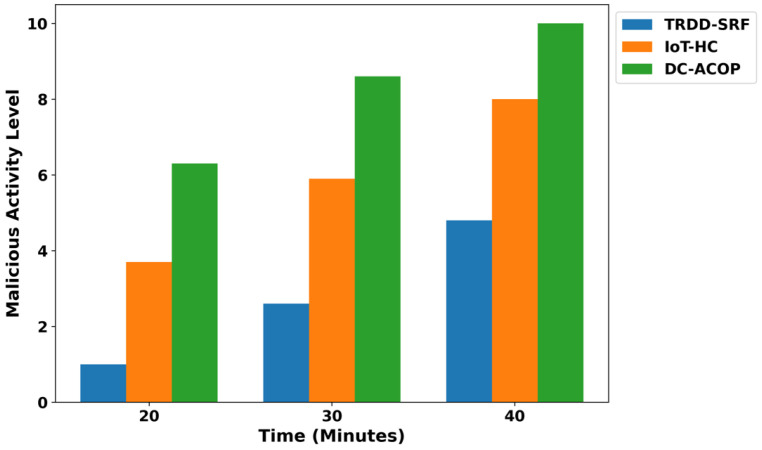
Malicious threats rate for varying health devices.

**Figure 11:**
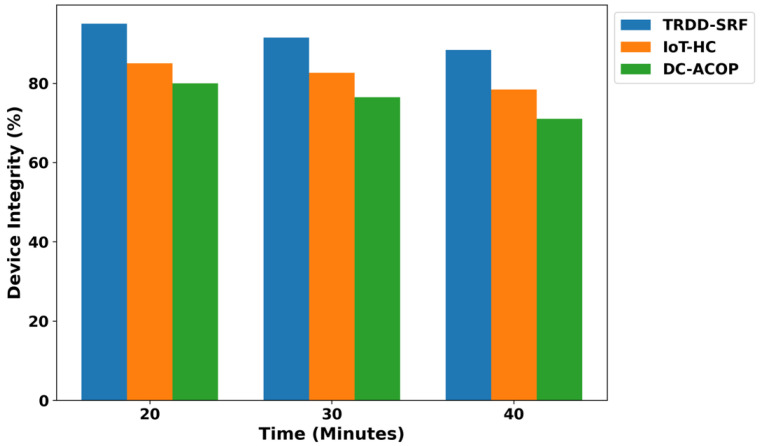
Threat detection time for varying health devices.

As shown in Figure 9, the TRDD-SRF model significantly improves the security threat level compared to existing models. By integrating trust evaluation mechanisms, the model reduces the security threat by 46% compared to IoT-HC and 52% compared to DC-ACOP. This is achieved through dynamic trust-based routing, which filters out unreliable devices and ensures secure communication. The model’s real-time device trust assessment ensures secure health data transmission, making it well-suited for real-time healthcare applications by exploring the machine learning classifier. In addition, the malicious devices are continuously authenticated through the integration of lightweight security approaches and are involved in the decision-making process. Figure 10 shows that the TRDD-SRF model also reduces malicious activity over varying time intervals. The primary role of the TRDD-SRF model is to establish trusted communication between health devices and eliminate high-risk connections based on the learning functionality of network conditions. Based on the results, it was noticed that the TRDD-SRF model lowers the malicious activity in the presence of fault devices by an average of 38% and 45%, respectively. It isolates the threatened devices and communication channels, ensuring data integrity and preventing disruptions in healthcare operations. In Figure 11, the performance of the device integrity is analyzed, and based on the results, a significant improvement of the TRDD-SRF model was revealed by an average of 47% and 51% in the comparison of related approaches. With varying times, the TRDD-SRF model maintains a higher percentage of trusted devices compared to IoT-HC and DC-ACOP, and enhances the authenticity of the medical devices for transmitting the health IoT data. At each time interval, the device integrity in the TRDD-SRF model is significantly higher, and it sustains its integrity by continuously assessing the role of devices to contribute to the reliability of the network. Moreover, the trusted behavior of the devices ensures a high level of accurate decision-making and identification of health disability among patients’ records.

## 5. Conclusions

By leveraging IoMT networks, healthcare applications have expanded to include continuous monitoring and instant data acquisition from the physical environment. The developed systems monitor real-time patient data for analysis and transmit it to cloud-level processing. It enables the development of automated decision-making for critical innovative medical applications and facilities for healthcare applications involving disability detection. However, due to the dynamic infrastructure of the IoMT systems, many approaches lack timely processing of large-scale data and inefficiently manage the system resources. In addition, securing insecure network channels, protecting against interaction with malicious traffic, safeguarding healthcare data, and ensuring authorized access are other significant research goals. Our proposed AI-driven model enhances the healthcare system by leveraging trusted and secure classification of disability patterns, utilizing the Random Forest algorithm. Specifically designed to manage large datasets related to critical health information and movement patterns, the model incorporates edge computing for intelligent learning. This approach enables accurate decision-making for disability detection and classifies abnormal movement patterns, while also isolating malicious behavior from IoMT devices. The model ensures the protection of the IoMT network, contributing to a more reliable and accessible healthcare system for individuals with disabilities. Moreover, through the collaborative interaction of devices in a trusted manner, the proposed model ensures a resilient infrastructure for effective and timely decision-making. However, the scalability of the proposed model can be enhanced by reducing the computational overhead of health sensors in distinguishing between normal and malicious traffic using more advanced deep-learning techniques. This enhancement can develop a more adaptive model for the rapid processing of medical requests, with improved resource management for real-time disability monitoring environments.

## Figures and Tables

**Table 1:** Summary of Existing Approaches and Contributions of the TRDD-SRF Model.

Existing Approaches	Contribution	Limitation	Significance of TRDD-SRF Model
Wearable IoMT Devices with Edge Computing [21,22]	Integration of wearable IoMT devices with edge computing for healthcare monitoring.	Limited focus on real-time disease detection and data trustworthiness.	Provides real-time disability detection by integrating IoMT sensors with a trust-based framework, improving both accuracy and security of the system.
Fault Detection in Biomedical Devices [27,28]	Non-invasive techniques for detecting defects in biomedical devices using edge-based anomaly detection.	Does not incorporate lightweight AI models in real-time patient monitoring.	Combines edge computing with Random Forest for real-time disability detection in healthcare, bridging the gap between structural fault detection and patient health monitoring.
Home Care Devices [31,32]	Development of home care devices with IoMT sensors for continuous health monitoring in non-clinical environments.	The focus is on sensor integration, with trustworthiness and real-time disease detection being overlooked.	Improves the system with trust-aware IoMT sensors, ensuring secure and reliable real-time feedback for home-based healthcare.
Optimization Techniques for IoT Healthcare Systems [33,34]	ACO-based routing for energy-efficient IoT healthcare systems.	Does not address privacy concerns or integrate AI classification for disease detection.	Enhances energy efficiency and privacy while integrating AI-based classification for disability detection, addressing privacy and trust issues.
Hybrid Modeling Approaches for Healthcare Applications [33,37]	Hybrid FEM-AI model for upper limb rehabilitation with real-time feedback.	Focused on rehabilitation, not disability detection in real-time healthcare systems.	Integrates real-time disability detection and edge computing, making it applicable to rehabilitation and home monitoring in healthcare settings.

**Table 2:** Key Characteristics of the Proposed TRDD-SRF Model for Adaptive Healthcare System.

Features	Description	Computing Contributions
Data Input	Raw sensor data from IoMT devices	Edge-based data collection for preprocessing
Preprocessing Techniques	Noise filtering, normalization, segmentation	Data processed at the edge to reduce latency
Feature Extraction Methods	Acceleration, angular velocity, postural angle	Real-time processing to minimize transmission
Trust Score Computation	Accuracy and freshness weighted sum	Security via trust evaluation for data integrity
Classification Method	Random Forest classifier	Edge-based classification reduces response time
Trusted Metrics	Normal/Abnormal detection	Trust-driven evaluation for accurate classification
Key Features of the Model	Edge preprocessing, trust evaluation, real-time detection	Efficient edge computing for reliable monitoring
Performance Metrics	Trust score, accuracy, precision, recall, F1-score	Metrics computed at edge to conserve bandwidth
Security Features	Trust score, malicious activity detection, thresholding	Trust-based evaluation of data and devices
Edge Device Utilization	Edge-level data processing	Lightweight edge solutions with enhanced security

**Table 3:** Security Analysis of Edge-Based IoMT Systems for Assistive Technology and Disability Detection.

Security Aspect	Description	Impact on Healthcare System
Data Integrity	Ensures accurate data through noise filtering and preprocessing ($D_{\mathrm{filtered}}$).	Protects against corrupted data, ensuring reliable health monitoring.
Confidentiality	Trust score computation filters unreliable data ($T_{\mathrm{trust}}$).	Safeguards patient privacy by excluding low-trust data ($T_{\mathrm{low}}$).
Authentication	Edge devices verify data authenticity before processing ($\alpha_{\mathrm{edge}}$).	Prevents unauthorized data or device manipulation, ensuring secure access.
Data Freshness	Trust score includes data freshness for timely health information ($T_{\mathrm{fresh}}$).	Ensures up-to-date data ($D_{\mathrm{fresh}}$) is used for timely decisions.
Access Control	Low-trust data are excluded based on trust score thresholds ($T_{\mathrm{threshold}}$).	Limits classification to trusted data ($T_{\mathrm{high}}$), reducing false diagnoses.
Malicious Activity Detection	Detects discrepancies in data quality during trust evaluation ($\delta_{\mathrm{anomaly}}$).	Mitigates risks from data manipulation or faulty sensors.
Data Transmission Security	Edge-based processing reduces data transmission ($D_{\mathrm{transmit}}$).	Minimizes exposure to breaches by reducing transmitted data volume.
System Resilience	Edge classification reduces reliance on cloud infrastructure ($C_{\mathrm{edge}}$).	Ensures continuous monitoring, even during network failures ($N_{\mathrm{failure}}$).

**Table 4:** Simulation Parameters.

Parameter	Value
Simulation Area	1000 m × 1000 m
Number of Sensors	100 to 300
Initial Energy per Node	5J
Simulation Run	70
Edge Devices	15
Malicious Devices	10 to 20
Packet Size	1024 bytes
Analyzing Scenarios	varying health devices
Performance metrics	energy consumption, packet drop ratio, and system resilience
Classification metrics	accuracy, precision, recall, and F1-score
Security analysis	threat level, malicious activity, and device integrity

## Data Availability

All data are available in the manuscript.

## References

[1] El-Saleh A.A., Sheikh A.M., Albreem M.A., Honnurvali M.S. (2025). The internet of medical things (IoMT): Opportunities and challenges. Wirel. Netw..

[2] Osama M., Ateya A.A., Sayed M.S., Hammad M., Pławiak P., Abd El-Latif A.A., Elsayed R.A. (2023). Internet of medical things and healthcare 4.0: Trends, requirements, challenges, and research directions. Sensors.

[3] Yang L., Amin O., Shihada B. (2024). Intelligent wearable systems: Opportunities and challenges in health and sports. ACM Comput. Surv..

[4] Yuan Y., Liu B., Li H., Li M., Song Y., Wang R., Wang T., Zhang H. (2022). Flexible wearable sensors in medical monitoring. Biosensors.

[5] Anikwe C.V., Nweke H.F., Ikegwu A.C., Egwuonwu C.A., Onu F.U., Alo U.R., Teh Y.W. (2022). Mobile and wearable sensors for data-driven health monitoring system: State-of-the-art and future prospect. Expert Syst. Appl..

[6] Cicirelli G., Impedovo D., Dentamaro V., Marani R., Pirlo G., D’Orazio T.R. (2021). Human gait analysis in neurodegenerative diseases: A review. IEEE J. Biomed. Health Inform..

[7] Buyya R., Ilager S., Arroba P. (2024). Energy-efficiency and sustainability in new generation cloud computing: A vision and directions for integrated management of data centre resources and workloads. Softw. Pract. Exp..

[8] Abughazalah M., Alsaggaf W., Saifuddin S., Sarhan S. (2024). Centralized vs. Decentralized Cloud Computing in Healthcare. Appl. Sci..

[9] Shukla S., Hassan M.F., Tran D.C., Akbar R., Paputungan I.V., Khan M.K. (2023). Improving latency in Internet-of-Things and cloud computing for real-time data transmission: A systematic literature review (SLR). Clust. Comput..

[10] Shahzad A., Chen W., Shaheen M., Zhang Y., Ahmad F. (2024). A robust algorithm for authenticated health data access via blockchain and cloud computing. PLoS ONE.

[11] Aminizadeh S., Heidari A., Dehghan M., Toumaj S., Rezaei M., Navimipour N.J., Stroppa F., Unal M. (2024). Opportunities and challenges of artificial intelligence and distributed systems to improve the quality of healthcare service. Artif. Intell. Med..

[12] Dhinakaran D., Ramani R., Edwin Raja S., Selvaraj D. (2025). Enhancing security in electronic health records using an adaptive feature-centric polynomial data security model with blockchain integration. Peer-to-Peer Netw. Appl..

[13] Adam M., Hammoudeh M., Alrawashdeh R., Alsulaimy B. (2024). A survey on security, privacy, trust, and architectural challenges in IoT systems. IEEE Access.

[14] Jaime F.J., Muñoz A., Rodríguez-Gómez F., Jerez-Calero A. (2023). Strengthening privacy and data security in biomedical microelectromechanical systems by IoT communication security and protection in smart healthcare. Sensors.

[15] Martínez A.L., Pérez M.G., Ruiz-Martínez A. (2023). A comprehensive model for securing sensitive patient data in a clinical scenario. IEEE Access.

[16] Neto E.C.P., Dadkhah S., Sadeghi S., Molyneaux H., Ghorbani A.A. (2024). A review of Machine Learning (ML)-based IoT security in healthcare: A dataset perspective. Comput. Commun..

[17] Arunprasath S., Annamalai S. (2024). Improving patient centric data retrieval and cyber security in healthcare: Privacy preserving solutions for a secure future. Multimed. Tools Appl..

[18] Almalawi A., Khan A.I., Alsolami F., Abushark Y.B., Alfakeeh A.S. (2023). Managing security of healthcare data for a modern healthcare system. Sensors.

[19] AlZubi A.A., Al-Maitah M., Alarifi A. (2021). Cyber-attack detection in healthcare using cyber-physical system and machine learning techniques. Soft Comput..

[20] Palimkar P., Shaw R.N., Ghosh A. (2021). Machine learning technique to prognosis diabetes disease: Random forest classifier approach. Advanced Computing and Intelligent Technologies: Proceedings of ICACIT 2021.

[21] Hartmann M., Hashmi U.S., Imran A. (2022). Edge computing in smart health care systems: Review, challenges, and research directions. Trans. Emerg. Telecommun. Technol..

[22] Ray P.P., Dash D., De D. (2019). Edge computing for Internet of Things: A survey, e-healthcare case study and future direction. J. Netw. Comput. Appl..

[23] Kamruzzaman M.M., Alanazi S., Alruwaili M., Alrashdi I., Alhwaiti Y., Alshammari N. (2022). Fuzzy-assisted machine learning framework for the fog-computing system in remote healthcare monitoring. Measurement.

[24] Rehman A., Saba T., Haseeb K., Larabi Marie-Sainte S., Lloret J. (2021). Energy-efficient IoT e-health using artificial intelligence model with homomorphic secret sharing. Energies.

[25] Nawaz Tareen F., Alvi A.N., Alsamani B., Alkhathami M., Alsadie D., Alosaimi N. (2024). EOTE-FSC: An efficient offloaded task execution for fog enabled smart cities. PLoS ONE.

[26] Jacobs M., He J., F. Pradier M., Lam B., Ahn A.C., McCoy T.H., Perlis R.H., Doshi-Velez F., Gajos K.Z. Designing AI for trust and collaboration in time-constrained medical decisions: A sociotechnical lens. Proceedings of the 2021 CHI Conference on Human Factors in Computing Systems.

[27] Versaci M., Angiulli G., Crucitti P., De Carlo D., Laganà F., Pellicanò D., Palumbo A. (2022). A fuzzy similarity-based approach to classify numerically simulated and experimentally detected carbon fiber-reinforced polymer plate defects. Sensors.

[28] Manimurugan S., Almutairi S., Aborokbah M.M., Narmatha C., Ganesan S., Chilamkurti N., Alzaheb R.A., Almoamari H. (2022). Two-stage classification model for the prediction of heart disease using IoMT and artificial intelligence. Sensors.

[29] Versaci M., Laganà F., Manin L., Angiulli G. (2025). Soft computing and eddy currents to estimate and classify delaminations in biomedical device CFRP plates. J. Electr. Eng..

[30] Manickam P., Mariappan S.A., Murugesan S.M., Hansda S., Kaushik A., Shinde R., Thipperudraswamy S.P. (2022). Artificial intelligence (AI) and internet of medical things (IoMT) assisted biomedical systems for intelligent healthcare. Biosensors.

[31] Menniti M., Laganà F., Oliva G., Bianco M., Fiorillo A.S., Pullano S.A. (2024). Development of Non-Invasive Ventilator for Homecare and Patient Monitoring System. Electronics.

[32] Mahmmod B.M., Naser M.A., Al-Sudani A.H.S., Alsabah M., Mohammed H.J., Alaskar H., Almarshad F., Hussain A., Abdulhussain S.H. (2024). Patient monitoring system based on internet of things: A review and related challenges with open research issues. IEEE Access.

[33] Rana B., Singh Y., Singh P.K., Hong W.C. (2024). A Priority Based Energy-Efficient Metaheuristic Routing Approach for Smart Healthcare System (SHS). IEEE Access.

[34] Lakshmanan R., Balakrishnan S., Mahendran A., Subramanian A.K. (2024). Multimodal healthcare data classification with Tangent Namib Beetle Optimization based routing in blockchain based IoT. Comput. Electr. Eng..

[35] Balakrishnan D., Rajkumar T.D., Dhanasekaran S., Murugan B.S. (2024). Secure and energy-efficient data transmission framework for IoT-based healthcare applications using EMCQLR and EKECC. Clust. Comput..

[36] Gupta S., Chithaluru P., Stephan T., Nafisa S., Kumar S. (2024). HSPBCI: A robust framework for secure healthcare data management in blockchain-based IoT systems. Multimed. Tools Appl..

[37] Laganà F., Pellicanò D., Arruzzo M., Pratticò D., Pullano S.A., Fiorillo A.S. (2025). FEM-Based Modelling and AI-Enhanced Monitoring System for Upper Limb Rehabilitation. Electronics.

[38] Kore A., Patil S. (2022). Cross layered cryptography based secure routing for IoT-enabled smart healthcare system. Wirel. Netw..

[39] Anguita D., Ghio A., Oneto L., Parra X., Reyes-Ortiz J.L. A public domain dataset for human activity recognition using smartphones. Proceedings of the 21st European Symposium on Artificial Neural Networks, Computational Intelligence and Machine Learning, ESANN.

